# Safety measures to prevent workplace violence in emergency primary care centres–a cross-sectional study

**DOI:** 10.1186/1472-6963-13-384

**Published:** 2013-10-03

**Authors:** Tone Morken, Ingrid H Johansen

**Affiliations:** 1 National Centre for Emergency Primary Health Care, Uni Health, Uni Research, Bergen, Norway; 2 Department of Global Public Health and Primary Care, University of Bergen, Bergen, Norway

## Abstract

**Background:**

Employees in emergency primary care centres (EPCC) have raised personal safety as an issue. Despite a high risk of experiencing workplace violence at EPCCs in Norway, knowledge regarding applied preventive measures is limited. The description of existing safety measures is an important prerequisite to evaluate and make guidelines for the improvement of preventive practices on a national level. The objective of this study was to investigate to which extent general practitioners work alone in EPCCs in Norway, and to estimate the prevalence of other preventive measures against workplace violence.

**Methods:**

A survey was sent to the managers of all 210 registered EPCCs in Norway. The questionnaire included 22 items on safety measures, including available staff, architecture and outfitting of the reception and consulting rooms, and the availability of electronic safety systems and training or monitoring systems. The data were analysed using descriptive statistics. Differences between EPCCs staffed by one general practitioner alone and EPCCs with more health personnel on duty were explored.

**Results:**

Sixty-one (30%) of the 203 participating EPCCs had more than one person on duty round-the-clock. These EPCCs reported the application of a significantly higher number of safety measures compared to the EPCCs with only one general practitioner on duty during some or part of the 24 hours. Examples of safety measures being more common in highly staffed EPCCs were automatic door locks (p < 0.001), arrangement of furniture in the consulting room ensuring that the patient is not seated between the clinician and the exit (p = 0.014), the possibility of bringing an extra person on emergency call-outs or home visits when needed for security reasons (p = 0.014), and having organised training regarding violence (p < 0.001).

**Conclusion:**

This study shows considerable differences between Norwegian EPCCs regarding applied preventive measures, and a higher prevalence of such measures in EPCCs staffed with several health personnel around-the-clock. More research is needed to understand the reasons for, and the effects of, these differences.

## Background

Employees in emergency primary care centres (EPCC) in Norway have raised concerns about personal safety and the lack of adequate security precautions to prevent violence in the workplace. Workplace violence can be defined as "incidents in which an employee is abused, sexually harassed, or assaulted in circumstances related to their work, involving an explicit or implicit challenge to their safety, well-being or health" [1]. A recent study has shown a high prevalence of workplace violence in Norwegian EPCCs: One in three employees has been physically abused during their career [2].

In Norway, the local municipalities are legally responsible for the 24/7 emergency medical services for all inhabitants [3]. During office hours, the emergency medical service is usually provided at general practitioners’ (GP) clinics, whilst EPCCs provide medical care during evenings, nights, weekends and public holidays. Some of the EPCCs are organised collaboratively between two or more municipalities. The number of staff present at any given time, including GPs (mandatory), nurses and other health personnel, varies between the EPCCs from one to more than ten persons on duty, from the smallest in the rural districts to the largest in the urban districts [4]. The GPs primarily treat patients at the centre, but they also carry out home visits and participate in on-site emergencies away from hospitals [5]. When nurses or other health personnel are present, they perform triage on patients who contact the centre, give advice when appropriate and assist the GP when needed.

Due to Norway’s strict two-tiered health care system, patients are not allowed to present themselves directly to hospital emergency departments. Out-of-hours they have to contact EPCCs when in need of medical aid. A referral from the EPCC’s GP is a prerequisite for access to secondary care. Patients self-refer to the EPCCs. As a matter of policy, EPCCs are therefore easily accessible to the public, and they handle all types of emergencies, including psychiatric emergency situations. The contact rates vary between the EPCCs from about 300 to 650 per 1000 inhabitants per year [6].

Emergency medicine and psychiatry have been singled out as high risk areas for experiencing workplace violence [7]. Despite the risk of workplace violence at EPCCs, there has been little focus on prevention. However, apprehension about violence has been found to be high in general practice [8] and in out-of-hours services in particular [9]. In a study of GPs’ experiences in Norwegian EPCCs, the GPs spontaneously raised personal safety as an issue [10]. They talked about problems with being alone and being completely left to their own device. They also reported low safety awareness at the centres.

Little is known regarding applied preventive measures. The description of existing safety measures is an important prerequisite to evaluate and make guidelines for the improvement of preventive practices on a national level. Thus, the objective of this study was to investigate to which extent GPs work alone in EPCCs in Norway, and to estimate the prevalence of applied measures to prevent workplace violence.

## Methods

In 2012, a web-based questionnaire was sent to the managers of each of the 210 registered EPCC in Norway. The questionnaire was part of an update of the National register for organisational data of all EPCCs in Norway, a register kept by request from The Norwegian Ministry of Health and Care Services. The register includes names of the manager for each of the EPCCs. Individual respondents (the managers) were thus identifiable. Every individual question on the online questionnaire had to be answered to proceed in the survey; hence there were no missing data in the completed questionnaires. The questionnaire was mainly about organisational information (management, staffing, equipment), and included a question regarding the application of 22 safety measures based on recommendations from the Norwegian Association of General Practitioners [11].

For analytic purposes, the 22-item list was grouped into five categories: (1) available staff, (2) reception, (3) consulting room, (4) electronic safety systems and (5) training/monitoring systems. The data were analysed using SPSS version 19. Differences in prevalence of safety measures between EPCCs with only one GP on duty and more highly staffed EPCCs were tested with Pearson chi-squared test. Statistically significance was accepted at p < 0.05.

The study was based on data from a survey used to update a national register, which is approved by the Norwegian Social Science Data Services. The survey involved organisational data only, and was not affected by the Norwegian Health Research Act. An approval from an ethics committee was therefore not needed [12].

## Results

A total of 203 (97%) of the 210 eligible EPCCs completed the questionnaire. Table 1 shows the prevalence of occupational safety measures applied.

**Table 1:** Prevalence of occupational safety measures in Norwegian emergency primary care centres (n = 203)

	**n**	**(%)**
***Available staff***		
Extra person on call-out/home visits when needed for security reasons	90	(44.3)
Personnel continuously accessible for patients/visitors	90	(44.3)
Always more than one person on duty	61	(30.0)
Security guard	7	(3.4)
***Reception***		
Barrier/glass partition between reception and waiting room	176	(86.7)
View to the waiting room	146	(71.9)
View to the entrance	125	(61.6)
Sheltered room	84	(41.4)
***Consulting room***		
Alternative exit	120	(59.1)
Quick entrance/exit for staff	93	(45.8)
Patient NOT sitting between clinician and door	59	(29.1)
***Electronic safety systems***		
Alarm on medical radio network	151	(74.4)
Automatic door lock	110	(54.2)
Panic button by desk/keyboard	76	(37.4)
Alarm linked to alarm center	62	(30.5)
Poster with information about alarm	59	(29.1)
Portable alarm	57	(28.1)
CCTV (closed-circuit television) camera	56	(27.6)
Panic button on wall	24	(11.8)
***Education and reporting system***		
Follow-up of employees after experienced violence	150	(74.9)
Monitoring system of violence against personnel	150	(74.9)
Training regarding violence against personnel	82	(40.4)

### Available staff

Only 30.0% of the EPCCs always had more than one person on duty. In less than half of the EPCCs (44.3%) an extra person could come on call-outs or home visits when needed for security reasons. Four EPPCs (2.0%) had applied every single measure in the available-staff-category, i.e. always more than one person on duty, personnel continuously accessible for patients/visitors, routines for an extra person on call-outs and home visits when needed for security reasons, and security guards always present. Sixty-eight centres (33.5%) had applied none of the four measures.

### Prevalence of other measures

The most frequently applied safety measure in the reception was a barrier or glass partition between the reception and the waiting room, reported by 176 centres (86.7%). This was also the most frequently applied of all 22 safety measures in question. A total of 54 centres (26.6%) reported that they had applied all measures related to the physical environment in reception areas, i.e. a barrier in place between reception and waiting room, a clear view to the entrance and the waiting room, and availability of sheltered rooms. Fourteen centres (6.9%) had none of these measures.

The most frequently applied measure related to the consulting room was the provision of an alternative exit, reported by 120 centres (59.1%). Thirty-eight centres (18.7%) had applied all measures, i.e. arrangement of furniture so that the patient did not sit between the clinician and the door, easily accessible entrance/exit for staff, and an alternative exit from the consulting room. Fifty-five centres (27.1%) had none of these recommended measures.

In the electronic equipment category the medical radio was the safety system most often used, applied by 151 centres (74.4%). One centre (0.5%) reported that all measures in this category were employed, including close-circuit television (CCTV) cameras, automatic door locks, alarms on the medical radio, panic buttons by the desk or keyboard, wall-mounted panic buttons, portable alarms, alarms linked to an alarm centre, and information posters about alarms. Nine centres (4.4%) employed none of these measures.

Most EPCCs stated that they had a system to follow-up employees who had experienced incidents of violence (n = 150, 74.9%). Seventy-five EPCCs (36.9%) had applied all measures regarding training and monitoring, including training regarding violence against personnel, incident reporting of episodes and follow-up of employees who had experienced incidents of violence. Thirty-five EPCCs (17.2%) reported that none of these measures were implemented.

### Differences between EPCCs with one on duty and EPCCs with more than one on duty

Most safety measures were significantly more prevalent at EPCCs which had more than one person present around the clock compared with the other EPCCs (Table 2). The only safety measure more frequently applied at EPCCs with only one GP on duty without support from other health personnel, was the use of the alarm on the medical network (p < 0.001).

**Table 2:** Occupational safety measures in EPCCs* with >1 person present (n = 61) compared to others (n = 142)

	**Always > 1 person on duty**				**p-value**
	**Yes**		**No**		
	**n**	**(%)**	**n**	**(%)**	
***Available staff***					
Extra person on call-out/home visits when needed for security reasons	35	(57.4)	55	(38.7)	0.014
Personnel continuously accessible for patients/visitors	55	(90.2)	35	(24.6)	<0.001
Security guard	5	(8.2)	2	(1.4)	N/A
***Reception***					
Barrier/glass partition reception – waiting room	56	(91.8)	120	(85.1)	0.192
View to the waiting room	52	(85.2)	94	(66.2)	0.006
View to the entrance	46	(75.4)	79	(56.0)	0.008
Sheltered room	34	(55.7)	50	(35.2)	0.005
***Consulting room***					
Alternative exit	39	(63.9)	81	(57.4)	0.360
Quick entrance/exit for staff	37	(60.7)	56	(39.4)	0.005
Patient NOT seated between clinician and door	25	(41.0)	34	(24.1)	0.014
***Electronic safety systems***					
Alarm on medical radio network	33	(54.1)	118	(83.1)	<0.001
Automatic door lock	45	(73.8)	65	(46.1)	<0.001
Panic button by desk/keyboard	29	(47.5)	47	(33.3)	0.051
Alarm linked to alarm center	27	(44.3)	35	(24.8)	0.005
Poster with information about alarm	19	(31.1)	40	(28.4)	0.668
Portable alarm	30	(49.2)	27	(19.1)	<0.001
CCTV (closed-circuit television) camera	34	(55.7)	22	(15.6)	<0.001
Panic button wall	16	(26.2)	8	(5.7)	<0.001
***Training*, *reporting and follow up***					
Follow-up of employees after violence	55	(90.2)	95	(67.4)	0.001
Monitoring system of violence against personnel	57	(93.4)	93	(65.5)	<0.001
Training regarding violence against personnel	37	(60.7)	45	(31.9)	<0.001

*EPCC: Emergency primary care centre.

## Discussion

To our knowledge, this is the first study on preventive measures in EPCCs in Norway. The study shows a high variation in the number of occupational safety measures applied. The reported variation in applied measures could reflect differences in perceived needs, due to differences in number or severity of violent incidents. However, it may also reflect a variation in organisational models, as larger organisations might allow more focus on administrative factors. This is further supported by the finding that implementation of most safety measures was significantly lower at EPCCs with only one GP present compared to EPCCs with more staff.

The study had a high response rate. The results are therefore likely to be representative of the managers of Norwegian EPCCs. However, because respondents did not retain anonymity, the validity of the study might have been compromised by managers trying to push a political statement in their response. As the 22-item list of measures was based on general recommendations from the Norwegian Association of General Practitioners, the list might include measures of limited relevance to the EPCCs. Furthermore, the list may lack essential preventive measures [13]. The effect of preventive measures on number of violent incidents is not known, and intervention studies are therefore needed. Nevertheless, our study gives an overview of the reported application of preventive measures, and the results provide insight into general preparedness regarding workplace violence.

Available staff is regarded as especially relevant as a safety measure to prevent workplace violence in EPCC. The presence of co-workers has been identified as a potential deterrent to assaults [14] and has also been recommended in an action plan proposal regarding organisation of emergency services in Norwegian primary health care [15]. It is therefore particularly worrying that the majority of GPs work alone for at least some of the time during opening hours, as this renders them more vulnerable should violent situations occur.

Some of the recommended measures related to the reception area and the consulting room are difficult to implement without changing the building structure. They must therefore be included in the initial planning phase of a new EPCC. Approximately one third of the EPCCs had none of the recommended measures regarding the consulting room. This suggests that prevention of workplace violence is often ignored in the process of planning or redesigning the EPCCs. The safety measure "patient NOT sitting between clinician and door" had the lowest prevalence in the consulting room category, though it might be rather easy to implement and possibly of high significance. Having a barrier or glass partition around the reception desk was common and can often be implemented without major structural changes. Nonetheless, in a low threshold service the application of barriers might create a potential conflict between ease of access, confidentiality and safety [16]. Due to the symbolic function of space and barriers, glass partitions might adversely affect patient-staff relationships, exacerbate violence and increase staff fearfulness [17,18]. This needs further exploration.

Monitoring systems are highly recommended to prevent occupational violence and consequences thereof [1,16,18], and seemed to be prevalent at the EPCCs. However, the preparation for negative incidents through training was less common. Violence-prevention training is deemed important and useful to prevent workplace violence in the health sector [18-20], and can probably be made available to all relevant staff independent of the size of the institution.

Having an alarm system to alert colleagues or others in the event of a problem is strongly recommended [18,19], and should possibly be a minimum recommendation. Four per cent of the EPCCs had no alert system, which seriously restricts the workers’ ability to summon help when needed. The reliance on the alarm on the national medical radio network, which is not primarily a safety tool, was more frequently reported among EPCCs with one GP working alone compared to other EPCCs. A previous study has shown that rural GPs use the medical radio network more frequently than their urban counterparts, and that they attend emergency situations more frequently than their urban colleagues [21]. In the smallest EPCCs the GP is based at home and attends the EPCC only when a consultation is needed. Some of the safety measures suggested in the 22-item list, such as security guard and personnel continuously accessible for patients/visitors, have low relevance for this type of organisation, and their absence might not be a safety risk for the GP. Nevertheless, focus on personnel safety is important independent of the organisation’s size, especially as these sole GPs are the most vulnerable should a violent incident occur.

## Conclusion

This study shows a considerable variation between Norwegian EPCCs regarding applied preventive measures, and a higher prevalence of such measures in EPCCs staffed with several health personnel around-the-clock. One should pay attention to the findings that in 70% of the EPPCs the GPs work alone for at least some of the time during opening hours, and that less than half of the EPCCs have systematic training regarding violence against personnel. More information is needed to judge the effectiveness of different measures and to make more appropriate recommendations. Given the variation in organisation and size of the EPCCs, we cannot assume that all suggested measures should be implemented everywhere. Even though some EPCCs have applied many preventive measures regarding workplace safety, we do not know if this makes any difference to the actual number of violent incidents or only to the subjective experience or consequences of the incidents. Further research should therefore focus on the experienced benefits of applied preventive measures.

## Competing interests

The authors declare that they have no competing interests.

## Authors’ contributions

Both authors participated in the design of the study. TM collected and analysed the data. Both authors drafted and edited drafts of the manuscript, and approved the final version.

## Pre-publication history

The pre-publication history for this paper can be accessed here:

http://www.biomedcentral.com/1472-6963/13/384/prepub
